# High-Fructose Corn-Syrup-Sweetened Beverage Intake Increases 5-Hour Breast Milk Fructose Concentrations in Lactating Women

**DOI:** 10.3390/nu10060669

**Published:** 2018-05-24

**Authors:** Paige K. Berger, David A. Fields, Ellen W. Demerath, Hideji Fujiwara, Michael I. Goran

**Affiliations:** 1 Department of Preventive Medicine, The University of Southern California, Los Angeles, CA 90089, USA; pberger@usc.edu; 2 Department of Pediatrics, Health Sciences Center, The University of Oklahoma, Oklahoma City, OK 73019, USA; David-Fields@ouhsc.edu; 3 Division of Epidemiology and Community Health, The University of Minnesota School of Public Health, Minneapolis, MN 55455, USA; ewd@umn.edu; 4 School of Medicine, Washington University in St. Louis, St. Louis, MO 63130, USA; hideji.fujiwara@gmail.com

**Keywords:** breast milk, breastfeeding, lactation, infant nutrition, infant feeding, sugar

## Abstract

This study determined the effects of consuming a high-fructose corn syrup (HFCS)-sweetened beverage on breast milk fructose, glucose, and lactose concentrations in lactating women. At six weeks postpartum, lactating mothers (*n* = 41) were randomized to a crossover study to consume a commercially available HFCS-sweetened beverage or artificially sweetened control beverage. At each session, mothers pumped a complete breast milk expression every hour for six consecutive hours. The baseline fasting concentrations of breast milk fructose, glucose, and lactose were 5.0 ± 1.3 µg/mL, 0.6 ± 0.3 mg/mL, and 6.8 ± 1.6 g/dL, respectively. The changes over time in breast milk sugars were significant only for fructose (treatment × time, *p* < 0.01). Post hoc comparisons showed the HFCS-sweetened beverage vs. control beverage increased breast milk fructose at 120 min (8.8 ± 2.1 vs. 5.3 ± 1.9 µg/mL), 180 min (9.4 ± 1.9 vs. 5.2 ± 2.2 µg/mL), 240 min (7.8 ± 1.7 vs. 5.1 ± 1.9 µg/mL), and 300 min (6.9 ± 1.4 vs. 4.9 ± 1.9 µg/mL) (all *p* < 0.05). The mean incremental area under the curve for breast milk fructose was also different between treatments (14.7 ± 1.2 vs. −2.60 ± 1.2 µg/mL × 360 min, *p* < 0.01). There was no treatment × time interaction for breast milk glucose or lactose. Our data suggest that the consumption of an HFCS-sweetened beverage increased breast milk fructose concentrations, which remained elevated up to five hours post-consumption.

## 1. Introduction

Breastfeeding is the optimal source of infant nutrition [1,2,3,4], however, there is a significant variation in breast milk both over time and between mothers because of a myriad of factors that affect its composition [5,6,7,8]. These compositional differences may be related to the maternal diet during lactation [7,8]. Because the current food environment affords the widespread availability of palatable, energy-dense, and inexpensive foods [9,10], and because there is limited research on the consequences of a “junk food” diet during lactation [11,12], there is a renewed interest in the effects of maternal intake on the properties of breast milk [13]. The modification of breast milk composition by maternal diet during lactation has the potential to impact infant growth and development [5,13,14].

Of particular interest to our group is the transmission of sugars from the mother’s diet to her breast milk, which can then be transmitted to the infant. We are specifically concerned with the effects of fructose. Early exposure to fructose has been shown to negatively affect appetite regulation, increase risk for obesity, and may set the foundation for the development of metabolic disease [15,16]. Our recent study was the first to report that fructose is present in breast milk at concentrations ranging from 4 to 8 µg/mL [13]. Despite these low concentrations, we showed that breast milk fructose was positively associated with infant body weight and body composition at age six months [13]. To our knowledge, no study has yet examined whether maternal intake of sugars during lactation alters the sugar content of breast milk, which, if documented, would indicate an additional avenue by which maternal intake affects infant nutritional status [17].

The primary aim of this randomized crossover study was to determine the acute effects of consuming a sugar-sweetened beverage that contained high-fructose corn syrup (HFCS; a mixture of free fructose and free glucose) on changes in breast milk fructose, glucose, and lactose concentrations in lactating women over 360 min (six hours) after consumption. An artificially sweetened beverage, which contained no caloric sugar, was used as a control. A secondary aim was to test moderation by pre-pregnancy obesity status.

## 2. Materials and Methods 

### 2.1. Subjects

Exclusively lactating women were enrolled at approximately six weeks postpartum. They were included in the study if they were 18 to 45 years old, had a pre-pregnancy body mass index (BMI) < 25.0 kg/m^2^ or >29.9 kg/m^2^ based on medical chart abstraction, had a singleton birth, and had a newborn ≥ 37 weeks gestational age. Exclusion criteria were as follows: (1) any tobacco use during gestation; (2) alcohol consumption defined as >1 drink per week containing 14 grams of ethanol; (3) diabetes; or (4) presumed or known congenital birth defects. All demographic information was collected by medical chart abstraction when possible. The University of Oklahoma Institutional Review Board approved all study procedures (clinicaltrials.gov registration #NCT02940795). Mothers provided informed consent for their participation in the study before the initiation of data collection.

### 2.2. Study Design

Of the 59 women screened for eligibility, 18 were excluded because they did not meet the inclusion criteria (*n* = 13), refused to participate (*n* = 2), and could not pump sufficient breast milk to be able to provide a sample at baseline (≥1 mL) (*n* = 3). In total, 41 mothers were randomized to the study. Nineteen were classified as normal weight (BMI < 25.0 kg/m^2^) and 22 were classified as obese (BMI > 29.9 kg/m^2^).

The enrolled participants were assigned sequentially to a computer-generated randomization list by the principal investigator (D.A.F.) to receive either the HFCS-sweetened beverage (Coca-Cola: Coca-Cola Company, Atlanta, GA, USA) or a control beverage (Diet Rite Cola: Dr. Pepper Snapple Group, Inc., Plano, TX, USA). Beverages were consumed on two separate days with a minimum of three days apart. The study participants and study personnel were not blinded to treatment order. Participants were provided with a hospital-grade breast pump (Symphony Breast Pump, Medela Inc., McHenry, IL, USA) and labeled bottles. Breast milk collection was conducted in the participants’ homes. To help ensure compliance with the protocol, participants visited the laboratory and received in-person instructions from trained study personnel. On the day prior to testing, participants were instructed to refrain from eating any candy, cookies, “junk food,” fruit, fruit juice, and soft drinks. After an overnight fast, participants pumped one complete breast milk expression as the baseline sample starting at 6:00 am. The HFCS-sweetened beverage (20 fluid ounces or 592 mL) contained 240 calories, 0 grams total fat, 75 mg sodium, 65 grams total carbohydrate (65 grams of sugar as HFCS-55), and 0 grams protein. The control beverage (12 fluid ounces or 355 mL) was artificially sweetened with acesulfame potassium and had no calories, total fat, sodium, total carbohydrate, and protein. The HFCS-sweetened beverage and the control beverage were consumed with a standard meal (Jimmy Dean Delights Turkey Sausage Breakfast Bowl: Tyson Foods, Inc., Springdale, AR, USA) provided by the study team. Participants were instructed to finish the contents of the beverage and meal within a 20-minute time frame.

Upon consuming the beverage and the contents of the standard meal, participants pumped one complete breast milk expression every hour for 6 consecutive hours. Per testing protocol, participants were instructed to immediately freeze each sample in accordance with proper handling procedures [18]. While participants were encouraged to pump from the right breast to standardize the testing protocol, they were allowed to use their discretion regarding which breast to pump from. Trained study personnel arranged to pick up frozen samples within two days of collection. The breast milk samples were placed on ice to take back to the University of Oklahoma Health Sciences Oklahoma City campus, where they were immediately frozen until batch analysis.

### 2.3. Breast Milk Analysis

#### 2.3.1. Preparation of Fructose Quantification Sample

The protein was removed by precipitating from the breast milk (50 µL) with addition of 200 µL of acetonitrile containing 10 µg of carbon-13 labeled (^13^C_6_)-fructose as the internal standard for natural fructose quantification in the breast milk. The supernatant, which contained natural fructose as well as ^13^C_6_-fructose, was collected after centrifugation of the protein precipitated breast milk for mass spectrometry analysis. The 5-point calibration samples were prepared for the absolute quantification of fructose in the breast milk. Quality control (QC) samples were also prepared by pooling some of the individual supernatants for the monitoring of analytical performance throughout the fructose analysis.

#### 2.3.2. Preparation of Glucose Quantification Sample

The serum protein was removed by precipitating from the breast milk (50 µL) with addition of 200 µL of acetonitrile containing 20 µg of deuterium labeled (d_2_)-glucose as the internal standard for natural glucose quantification in the breast milk. The supernatant, which contained natural glucose as well as glucose- d_2_, was collected after centrifugation of the protein precipitated breast milk for MS analysis. The 4- or 5-point calibration samples were prepared for the absolute quantification (mg/mL) of glucose in the breast milk. QC samples were also prepared by pooling some of the individual supernatants for the monitoring of analytical performance throughout the glucose analysis.

#### 2.3.3. Preparation of Lactose Quantification Sample

The original breast milk was initially diluted 100-fold with water. Then, 5 µL of the diluted breast milk was again diluted by 200-fold with 995 µL of water containing 5 µg of ^13^C_12_-lactulose as the internal standard. The 4-point calibration samples were prepared for the absolute quantification of lactose in the breast milk. QC samples were prepared for lactose analysis.

#### 2.3.4. Liquid Chromatography-Mass Spectrometry Analysis

The fructose, glucose, and lactose analyses were performed with a Shimadzu 20AD HPLC system and a Leap PAL autosampler coupled to a triple quadrupole mass spectrometer (API-4000: Applied Biosystems, Foster City, CA, USA) operated in MRM mode, as described by Goran et al. [13].

### 2.4. Statistical Analyses

#### 2.4.1. Sample Size Calculation

Power calculations were computed based on the primary outcome breast milk fructose, glucose, and lactose. Assumptions are required concerning the difference in mean changes in the response (i.e., differences in the mean breast milk fructose, glucose, and lactose values) across treatment groups, and the variance of the gain score, from which effect size can be computed. Using data previously collected in our laboratory [13], we pooled estimates of the standard deviation (SD) of change for breast milk fructose (SD = 0.2 µg/mL), glucose (SD = 0.1 mg/mL), and lactose (SD = 0.2 mg/mL), and we estimated effect sizes (i.e., Cohen’s *d*) for these outcomes (fructose, *d* = 0.57; glucose, *d* = 0.48; and lactose, *d* = 0.76) based on the assumption that the HFCS-sweetened beverage would elicit at least a 10% mean change difference relative to the control beverage. We determined that 18 to 37 subjects/group would provide 80 to 85% power (α = 0.05) to detect a difference in the mean change of the outcome variable between treatments. With a given sample size of 37 subjects/group (α level set at 0.05), the proposed study had a power of 0.80. Assuming a 10% sample size loss due to either attrition or the insufficient quality of measurements, a starting sample size of 41 subjects per treatments preserved the power of the study design.

#### 2.4.2. Analyses

Group differences in the baseline characteristics between normal weight and obese participants were determined by unpaired *t*-tests for continuous variables and *χ*^2^ tests for categorical variables. Differences in the concentrations of breast milk fructose, glucose, and lactose by treatment over the 6-hour testing period were determined using a 2-factor repeated measures analysis of variance (ANOVA) with the main effects of treatment and time and treatment × time interaction with repeated measures for the participants after the HFCS-sweetened beverage and control beverage challenges, respectively. If the treatment × time interaction was significant at *p* < 0.05, multiple comparisons at each time point were performed using the Bonferroni method. Covariance structures were compared using the Akaike information criterion; the autoregressive structure and the compound symmetry structure had similar Akaike information criterion values, so the compound symmetry structure was chosen. To test moderation by pre-pregnancy obesity status, a 3-factor repeated measures ANOVA was performed for each outcome measure in separate models. In addition, calculations for the incremental area under the curve (iAUC) were performed for breast milk fructose, glucose, and lactose using the trapezoidal method. To assess differences in iAUC for breast milk fructose, glucose, and lactose in response to the beverages, independent sample *t* tests were conducted. All statistical analyses were performed using SPSS software (version 22.0, 2013: IBM SPSS Statistics, Armonk, NY, USA). A *p*-value < 0.05 was considered statistically significant in all analyses.

## 3. Results

**Figure 1:**
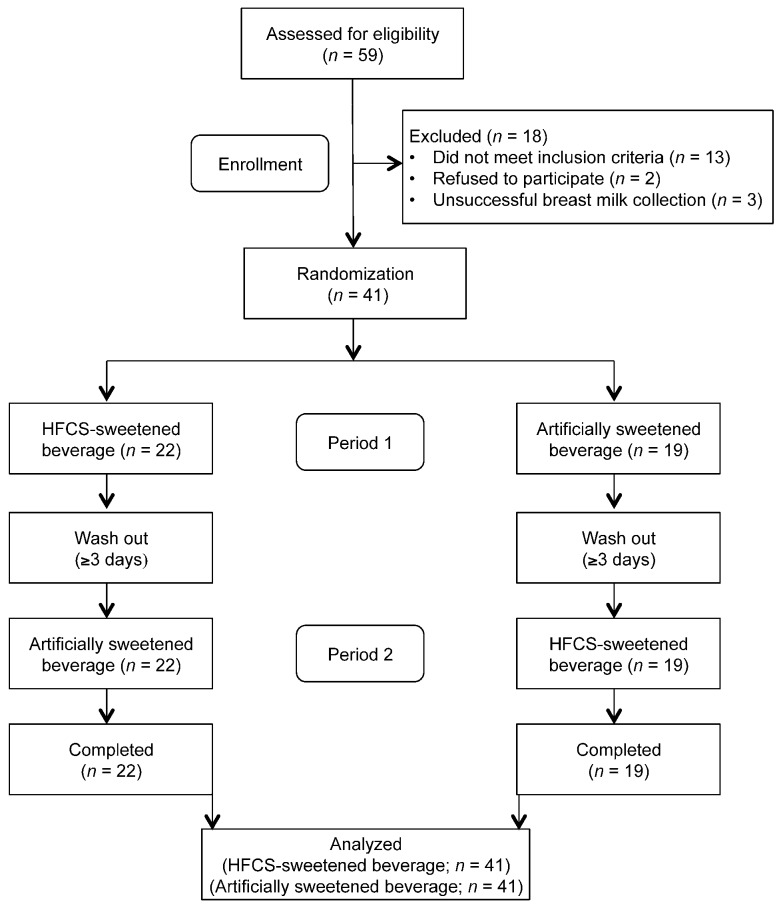
Flow diagram of participants.

The flow diagram of the participants is presented in Figure 1. Of the 59 women screened for eligibility, 69% (41 women) met the inclusion criteria. The baseline characteristics of the participants by pre-pregnancy obesity status are presented in Table 1. By design, the normal weight group had a lower BMI compared to the obese group (23.1 ± 1.7 kg/m^2^ vs. 33.7 ± 4.2 kg/m^2^, *p* < 0.01). However, the fasting baseline concentrations of breast milk fructose, glucose, and lactose did not differ between pre-pregnancy obesity status groups.

**Figure 2:**
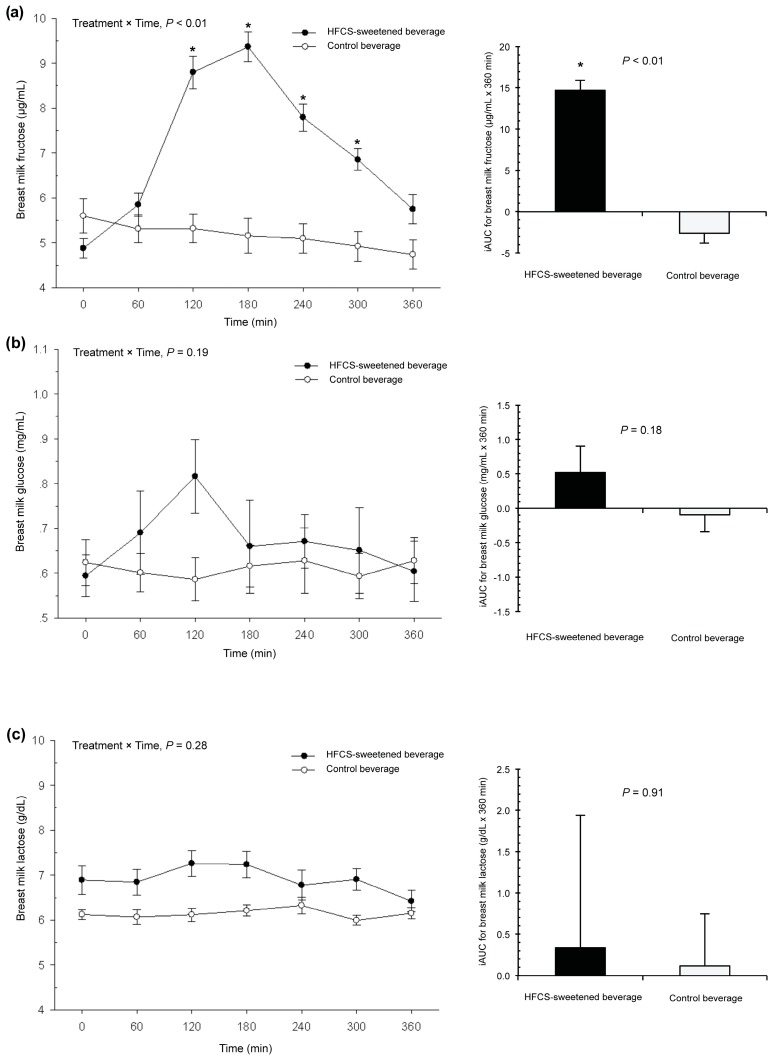
Breast milk concentrations of (**a**) fructose, (**b**) glucose, and (**c**) lactose in 41 lactating women after consumption of a HFCS-sweetened beverage or control beverage. Values are mean ± SEM. HFCS, high-fructose corn syrup; iAUC, incremental area under curve; SEM, standard error of mean.

Figure 2a shows the breast milk fructose response to each beverage. The fasting baseline fructose concentration was 5.0 ± 1.3 µg/mL. There was a significant increase in the baseline concentrations of breast milk fructose in response to the HFCS-sweetened beverage, but not the control beverage (treatment × time interaction, *p* < 0.01). For the HFCS-sweetened beverage arm, the time to peak breast milk fructose was 180 min (three hours) and the peak concentration was 9.4 ± 1.9 µg/mL. Concentrations remained significantly higher than the baseline up to 300 min (five hours) after consumption (*p* < 0.01). The mean iAUC was also significantly higher in response to the HFCS-sweetened beverage than the control beverage (14.7 ± 1.2 vs. −2.60 ± 1.2 µg/mL × 360 min, *p* < 0.01). There was no moderation by pre-pregnancy obesity status.

Figure 2b shows the breast milk glucose response. The fasting baseline glucose concentration was 0.6 ± 0.3 mg/mL. There was no change in the baseline concentrations of breast milk glucose in response to the HFCS-sweetened beverage compared to the control beverage (treatment × time interaction, *p* = 0.19), and there were no differences in mean iAUC between groups (*p* = 0.18). Figure 2c reveals the findings for breast milk lactose. The fasting baseline lactose concentration was 6.8 ± 1.6 g/dL. There was no treatment × time interaction for concentrations of breast milk lactose (*p* = 0.28), and there were no differences in the mean iAUC between groups (*p* = 0.91). In addition, there was no moderation by pre-pregnancy obesity status for glucose or lactose responses.

## 4. Discussion

This is the first study that we are aware of a report that consuming an HFCS-sweetened beverage during lactation leads to an increase in fructose concentrations, but not glucose or lactose concentrations, in breast milk. Studies to date have found that variability in maternal diet and weight status both contribute to heterogeneity in breast milk composition [7,8,19]. Our objective was to test whether the consumption of an HFCS-sweetened beverage had any impact on breast milk sugar composition. In a group of 41 lactating women, we found that consuming 20 fluid ounces (592 mL) of an HFCS-sweetened beverage (65 grams of sugar as HFCS-55) compared to an artificially sweetened beverage (zero grams of sugar) doubled the concentration of fructose in breast milk within three hours, regardless of pre-pregnancy BMI, and remained elevated for five hours. Though the fructose concentrations were much lower than those of glucose and lactose, our work to date suggests that this may still be biologically relevant for infants and warrants further investigation [13,20].

Our findings confirmed those of our previous work on a separate cohort of women, that fructose is detectable in breast milk and was apparent at a “basal” concentration of approximately 5 µg/mL [13]. To build on this, we provide new evidence in the current study that fructose in breast milk can be derived from fructose in the maternal diet. When normal weight and obese mothers consumed 20 fluid ounces (592 mL) of an HFCS-sweetened beverage, which may contain 36 to 40 grams of fructose, depending on the HFCS formulation [21], the concentration of breast milk fructose increased by 92% within three hours relative to the control beverage. Concentrations were still significantly higher than baseline for five hours after consumption. This suggests the potential for the elevation of fructose if mothers regularly consume HFCS-sweetened products in the hours before and during breastfeeding. Because infants tend to feed every two to three hours in the first month, they could be well within the window for multiple exposures to fructose. 

During the HFCS-sweetened beverage challenge, concentrations of breast milk fructose peaked at 9.4 µg/mL. We recognize that this amount of fructose is quite low and may not even exert any biological effects on infants due to the underdeveloped fructose transporter, GLUT5 [22]. On the other hand, there is evidence to suggest that it might be clinically relevant during this critical window of development. For example, data have shown that a dose similar to our peak fructose concentration (i.e., 10 µg/mL) primed pre-adipocyte precursor cells for fat tissue growth [23]. Moreover, we recently reported that a mean of 7 µg/mL of breast milk fructose was associated with greater infant body weight and body composition (fat mass, lean mass, and bone mass) at age six months [13]. Because fructose from the maternal diet is metabolized by the liver, repackaged into triglycerides, and may be released into circulation [24,25], one potential hypothesis is that triglycerides spill over into breast milk and affect infant growth [26,27]. However, more work is needed to examine how fructose is metabolized in lactating women and the potential mechanisms that may underlie our findings.

In contrast, there was no significant effect of either beverage on the concentrations of glucose or lactose in breast milk. This is in agreement with findings from other studies, namely that changes in maternal diet are thought to elicit no changes in the principal sugars in breast milk [7,8]. The differential effects of an HFCS-sweetened beverage on the concentrations of breast milk fructose vs. glucose could be attributed to the distinct ways in which they are used in the body. While fructose is metabolized exclusively in the liver and is not well regulated [28], glucose is metabolized in all organs and is tightly controlled by insulin. Insulin may regulate blood glucose concentrations before there is an opportunity for them to enter the mammary epithelial cells via a passive, facilitated process driven by the downward glucose concentration gradient [29,30,31]. Circulating fructose concentrations, however, are not subject to the same regulated process to enter breast milk [31,32]. Accordingly, the amount of breast milk fructose would be directly related to the amount of fructose consumed in the diet.

This study is not without limitations. First, the sample was relatively small and racially and demographically homogenous, which limits the generalizability of our findings. Another potential limitation was that breast milk collection was conducted in the participants’ homes, and information regarding the adherence to study protocol (e.g., refraining from sugar-laden products) was not obtained. We did not have dietary information to describe the habitual consumption of sugar. It is also important to note that we did not assess biochemical parameters indicative of metabolic abnormalities in breast milk (i.e., insulin) or blood (i.e., glucose). Lastly, our study was not statistically powered to detect a moderation effect by pre-pregnancy obesity status. It would be worthwhile to explore this further in appropriately powered trials due to the metabolic implications of obesity [30]. 

## 5. Conclusions

Overall, the findings from this study revealed that mothers’ consumption of an HFCS-sweetened beverage significantly increased concentrations of breast milk fructose, which persisted for five hours, with no effect on breast milk glucose or lactose. The consequences of the habitual elevation of breast milk fructose for infants require further investigation [13,15,20]. This should be explored with a larger sample size that collects additional data on maternal diet as well as metabolic outcomes to better understand the potential implications of foods and beverages sweetened with fructose-containing sugars in early life.

## Figures and Tables

**Table 1:** Baseline characteristics of the participants at six weeks postpartum. ^1^

	Total	Normal Weight	Obese	*p* ^2^
*n*	41	19	22	
Maternal age (years)	29.8 ± 2.9	30.5 ± 2.6	29.3 ± 3.0	0.21
Whites (%) ^3^	91	100	83	0.40
Pre-pregnancy weight (kg)	79.0 ± 19.7	62.0 ± 7.3	91.4 ± 16.2	<0.01
Pre-pregnancy height (cm)	163 ± 6.9	164 ± 5.8	163 ± 7.9	0.77
Pre-pregnancy BMI (cm)	29.2 ± 6.3	23.1 ± 1.7	33.7 ± 4.2	<0.01
Infant age (days)	35.5 ± 3.5	35.6 ± 3.2	35.4 ± 4.3	0.92
Baseline breast milk sugars				
Fructose (µg/mL)	5.04 ± 1.3	5.21 ± 1.3	4.92 ± 1.3	0.52
Glucose (mg/mL)	0.64 ± 0.3	0.67 ± 0.4	0.63 ± 0.2	0.75
Lactose (g/dL)	6.83 ± 1.6	7.00 ± 1.1	6.72 ± 1.8	0.65

^1^ Values are means ± SD or %; ^2^ Tests of significance between groups were based on independent sample *t* tests, unless otherwise indicated; ^3^ Tests of significance between groups were based on chi-square tests.
